# Tumor Microenvironment and Immune Response Against Wilms Tumor: Evasion Mechanisms and Implications for Immunotherapeutic Approaches

**DOI:** 10.3390/cancers18060908

**Published:** 2026-03-11

**Authors:** Claudia Cantoni, Valerio Gaetano Vellone, Barbara Cafferata, Gabriele Gaggero, Martina Serra, Filippo Spreafico, Cristina Bottino, Grazia Maria Spaggiari

**Affiliations:** 1 Department of Experimental Medicine (DIMES), University of Genoa, 16132 Genoa, Italy; claudia.cantoni@unige.it (C.C.); martina.serra@unige.it (M.S.); grazia.maria.spaggiari@unige.it (G.M.S.); 2 Clinical and Experimental Immunology Unit, Department of Services, IRCCS Istituto Giannina Gaslini, 16147 Genoa, Italy; 3 Pathology Unit, IRCCS Istituto Giannina Gaslini, 16147 Genoa, Italy; barbaracafferata@gaslini.org (B.C.); gabrielegaggero@gaslini.org (G.G.); 4 Department of Integrated Surgical and Diagnostic Sciences (DISC), University of Genoa, 16132 Genoa, Italy; 5 Oncology Unit, IRCCS Istituto Giannina Gaslini, 16147 Genoa, Italy; filippospreafico@gaslini.org

**Keywords:** Wilms tumor, tumor microenvironment, tumor escape, natural killer cells, macrophages, immunotherapy, targeted therapy

## Abstract

Wilms tumor (WT) is the most common malignant renal tumor in childhood and represents one of the major success stories of pediatric oncology, with very good survival achieved through risk-adapted multimodal therapy. Nevertheless, a subset of patients—particularly those with diffuse anaplasia, blastemal-type tumors persisting after chemotherapy, or relapsed disease—continues to experience poor outcomes and significant long-term treatment-related morbidity. These challenges highlight the need for novel therapeutic strategies beyond conventional cytotoxic approaches. Growing evidence indicates that WT is characterized by a complex and distinctive tumor microenvironment (TME) shaped by its developmental origin and triphasic histology. Immune cell infiltration, inflammatory mediators, and immune checkpoint pathways interact differently with blastemal, epithelial, and stromal tumor components, generating heterogeneous immune surveillance and escape mechanisms. In particular, tumor-associated macrophages (TAMs), functionally impaired natural killer (NK) cells, and immunosuppressive stromal elements play a central role in shaping an immune milieu that may limit the efficacy of immune-based therapies. Although immunotherapy has changed the management of several adult malignancies and some pediatric cancers, its translation to WT has so far been limited, with modest results in unselected patient populations. Recent immunogenomic and proteogenomic studies, however, suggest the existence of biologically distinct WT subsets with different immune features and potential susceptibility to targeted immunotherapeutic approaches. This narrative review integrates pathological, immunological, and clinical perspectives to summarize current knowledge on the WT immune microenvironment, mechanisms of tumor immune evasion, and emerging immunotherapeutic strategies. By providing a unified framework, it aims at supporting a multidisciplinary approach for the rational development of future immune-based and combination therapies tailored to specific WT subgroups.

## Simple Summary

Wilms tumor (WT) is the most common kidney cancer in children, which is usually successfully treated with surgery and chemotherapy. However, some children develop aggressive or recurrent disease that does not respond well to standard treatments and may cause long-term side effects. Although immunotherapy, which helps the immune system to recognize and attack cancer cells, has improved outcomes in many adult cancers in recent years, its possible employment and efficacy in WT is still under investigation. This review explains how the immune system interacts with WT, how cancer cells can escape immune control, and why current immunotherapies have shown limited benefit so far. By combining pathological, biological, and clinical perspectives, this paper highlights new opportunities for immune-based treatments and the importance of a multidisciplinary approach to improve care for children with high-risk WT.

## 1. Introduction

Wilms tumor (WT) is the most frequent malignant renal tumor occurring in childhood and one of the major success stories of pediatric oncology, with survival rates exceeding 90% in most patients treated with multimodal, risk-adapted protocols [1]. Nevertheless, a clinically relevant subset of children—particularly those with diffuse anaplasia, blastemal-type tumors after chemotherapy, or relapsed disease—continues to experience poor outcomes and substantial long-term treatment-related toxicity. These unmet clinical needs highlight the need for biologically informed therapeutic strategies beyond conventional cytotoxic approaches.

Increasing evidence indicates that WT is not immunologically inert but, rather, characterized by a complex and peculiar tumor microenvironment (TME) shaped by its developmental origin and triphasic histology. Distinct immune cell populations, inflammatory mediators, and immune checkpoint pathways interact differently with blastemal, epithelial, and stromal tumor components, contributing to heterogeneous immune surveillance and escape mechanisms. These features provide a strong biological rationale for exploring immunotherapeutic strategies, while simultaneously explaining the limited efficacy observed with available immune checkpoint inhibitors and other immune-based treatments in unselected WT populations.

By providing a unified framework, this review seeks to support a multidisciplinary perspective and to inform the rational design of future immune-based and combination therapies tailored to specific biological subsets of WT.

This narrative review was conducted according to recommended standards for high-quality narrative synthesis and guided by the SANRA (Scale for the Assessment of Narrative Review Articles) framework [2]. A comprehensive literature search was performed using PubMed, Scopus, and Web of Science, complemented by targeted manual searches of references from key publications and international clinical guidelines issued by the Children’s Oncology Group (COG) and the International Society of Paediatric Oncology (SIOP). Peer-reviewed articles, reviews, and consensus or practice documents published in English between 2000 and 2025 were considered eligible.

### 1.1. Epidemiology, Risk Factors, and Outcomes of WT

Despite its relatively uniform histology, WT shows marked global variation in incidence, demographics, and survival, reflecting the combined effects of genetic susceptibility, perinatal exposures, and healthcare system disparities.

WT predominantly affects very young children, with its incidence peaking between birth and four years of age at approximately 15.1 cases per million, followed by a sharp decline [1]. In high-income countries such as the United States, WT accounts for about 5% of pediatric cancers and up to 7% overall [3]. Its sex distribution is generally balanced, with a slight female predominance in most regions except Eastern Asia [3]. Significant racial and ethnic disparities are observed, with children of sub-Saharan African ancestry presenting the highest reported incidence (approximately 11 cases per million), exceeding rates in White and East Asian populations [4]. These differences likely reflect both biological predisposition and social determinants of health.

Although WT is primarily a genetically driven embryonal tumor, environmental and perinatal factors may modulate risk. A 2024 systematic review and meta-analysis of 58 studies identified associations with high birthweight, large-for-gestational-age status, prematurity, cesarean delivery, and parental (particularly maternal) occupational pesticide exposure [5]. Breastfeeding and maternal folic acid or multivitamin supplementation were associated with reduced risk, while no convincing associations were found for parental age, smoking, alcohol use, or maternal diagnostic X-ray exposure. Overall, these factors exert modest effects compared with genetic susceptibility.

Approximately 10% of WT cases occur in association with congenital anomalies or hereditary cancer predisposition syndromes, including Beckwith–Wiedemann syndrome, WAGR syndrome, Denys–Drash syndrome, and isolated hemihypertrophy [6]. These conditions involve pathogenic variants or epigenetic alterations affecting key developmental loci, notably *WT1* (11p13), *WT2* (11p15.5), and *DIS3L2*.

### 1.2. Genetic Landscape of WT

WT displays a highly heterogeneous genetic architecture despite a relatively low overall somatic mutational burden. Genomic studies have identified alterations in approximately 40 cancer-associated genes—many involved in fetal kidney development—supporting WT as a prototypical embryonal malignancy arising from disrupted renal organogenesis rather than progressive accumulation of random mutations [7]. While about 10% of cases occur in defined genetic predisposition syndromes, most WTs are sporadic and driven by somatic mutations, copy number changes, and epigenetic alterations converging on a limited set of developmental pathways.

Germline susceptibility is central in syndromic WT. Constitutional *WT1* alterations underlie WAGR and Denys–Drash/Frasier syndromes, biallelic *DIS3L2* mutations cause Perlman syndrome, and epigenetic or structural abnormalities at 11p15 define the Beckwith–Wiedemann spectrum. Germline variants in *TRIM28*, *REST*, *CHEK2*, *PALB2*, and *BRCA2* have also been implicated in familial or nonsyndromic WT [7,8]. These alterations often represent the first oncogenic “hit” and are associated with bilateral or multifocal disease and earlier onset.

At the somatic level, three major pathways dominate WT biology. The WT1-Wnt/β-catenin axis is foundational, with *WT1* mutations occurring in 10–15% of tumors and frequently co-occurring with 11p15 abnormalities and activating *CTNNB1* mutations, indicating functional cooperation between nephron progenitor specification and WNT signaling [7,8]. Inactivation of *WTX/AMER1* further underscores the centrality of β-catenin dysregulation. The 11p15 (“*WT2*”) locus represents a second major driver. Loss of imprinting or heterozygosity results in *IGF2* overexpression and/or *H19* silencing, which is among the most frequent events in WT and is particularly enriched in stromal-predominant tumors and those associated with the Beckwith–Wiedemann spectrum [7]. Transcriptomic profiling confirms near-universal *IGF2* overexpression in blastemal-type tumors [9].

A third defining mechanism involves disruption of microRNA (miRNA) processing. Somatic mutations in *DROSHA*, *DGCR8*, *DICER1*, and related genes occur in up to 15–20% of blastemal-type WTs, leading to global miRNA depletion and persistence of an undifferentiated, self-renewing progenitor state [7,9].

Additional driver events affect transcription factors and chromatin regulators that are essential for nephrogenesis. Recurrent *SIX1* and *SIX2* hotspot mutations—particularly Q177R—are present in nearly 18% of blastemal-type tumors and correlate with proliferative signatures and adverse outcome [9]. Alterations in epigenetic regulators, including *BAF*/*SWI-SNF* complex members, histone modifiers, and MYCN-interacting proteins, further reinforce WT as a malignancy of arrested renal differentiation [7].

Copy number alterations add prognostic significance. Combined loss of heterozygosity at 1p and 16q is an established adverse biomarker in Children’s Oncology Group protocols, while 11p15 abnormalities refine risk stratification [8]. Gain of chromosome arm 1q, present in approximately 20–30% of cases, has emerged as the most robust predictor of poor outcome, independently associated with inferior event-free and overall survival across multiple cohorts [10,11,12].

### 1.3. Histopathology of WT

Wilms tumor (WT) is a prototypical embryonal malignancy of the developing kidney. As outlined in the preceding epidemiologic and genetic sections, WT arises from disrupted nephrogenesis; histopathology represents the morphologic counterpart of these alterations and remains central to diagnosis, staging, risk stratification, and therapeutic decision-making in both COG and SIOP protocols [13,14].

**Figure 1:**
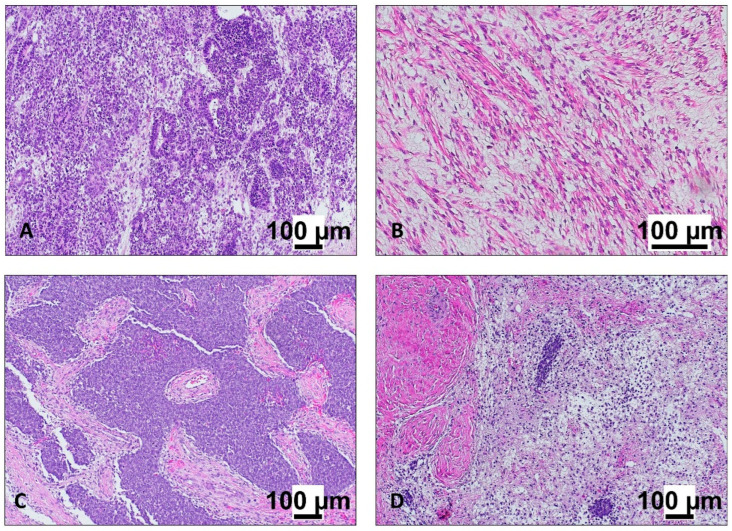
Histopathologic patterns of Wilms tumor. Representative hematoxylin and eosin-stained sections at original magnification ×100 illustrating the principal histologic components and therapy-related changes in Wilms tumor. (**A**) Epithelial component, composed of primitive tubules and glomeruloid structures lined by cuboidal to columnar cells, recapitulating abortive nephron differentiation. (**B**) Stromal component, characterized by spindle-shaped mesenchymal cells within a myxoid to fibrous matrix, occasionally showing features of mesenchymal differentiation. (**C**) Blastemal component, showing solid sheets and nodules of undifferentiated small round blue cells with a high nuclear-to-cytoplasmic ratio and brisk mitotic activity. (**D**) Post-therapy changes, with prominent fibrosis, hyalinization, inflammatory infiltrates, and scattered residual viable tumor elements, consistent with chemotherapy-induced regression.

Macroscopically, WT typically presents as a large, soft, tan-gray, lobulated renal mass with frequent hemorrhage, necrosis, and cystic change. Microscopically, classic favorable-histology WT shows a triphasic pattern of blastemal, epithelial, and stromal components in variable proportions (Figure 1) [13,15]. Blastemal areas consist of small round blue cells with high mitotic activity; epithelial components form primitive tubules or glomeruloid structures; stromal elements show mesenchymal differentiation and may include heterologous tissues such as skeletal muscle, cartilage, bone, adipose tissue, or smooth muscle. Many tumors deviate from the classic triphasic pattern and may be monophasic or biphasic, underscoring the need for extensive sampling, as minor components may be diagnostically and prognostically relevant [13]. Nephrogenic rests, which are foci of persistent embryonal renal tissue, are recognized precursor lesions and are frequently identified in kidneys affected by WT. They are classified as perilobar or intralobar, with diffuse involvement termed nephroblastomatosis. Perilobar rests are strongly associated with the Beckwith–Wiedemann spectrum, whereas intralobar rests are more common in *WT1*-related syndromes such as WAGR and Denys–Drash, linking histopathology with genetic predisposition mechanisms [14,15].

Histologic classification differs between COG and SIOP paradigms, reflecting differences in treatment sequencing. In COG protocols, which favor upfront nephrectomy, tumors are classified as favorable histology or anaplastic WT, with therapy guided mainly by stage, age, tumor weight, and selected molecular features [16]. Accurate recognition of epithelial-, stromal-, or blastemal-predominant patterns remains important for appropriate treatment assignment. In contrast, SIOP protocols assess histology after preoperative chemotherapy, categorizing tumors into low-, intermediate-, or high-risk groups based on residual viable components and treatment response. Blastemal-type tumors persisting after chemotherapy and diffuse anaplastic tumors define the high-risk category and are associated with significantly worse outcomes [17].

Anaplasia is the most important adverse histologic feature in WT. Defined by marked nuclear enlargement, hyperchromasia, and abnormal multipolar mitoses, it is classified as focal or diffuse. Diffuse anaplasia is associated with chemoresistance and poor prognosis, whereas focal anaplasia may behave similarly to favorable-histology tumors when completely resected [18]. Because anaplasia may be focal, meticulous sampling is essential. To standardize reporting, the International Collaboration on Cancer Reporting (ICCR) has developed a synoptic dataset for pediatric renal tumors, mandating uniform documentation of tumor type, component proportions, nephrogenic rests, invasion patterns, margins, lymph nodes, and treatment effect, thereby harmonizing reporting across COG and SIOP frameworks [19].

In summary, histopathology integrates developmental biology, genetics, and clinical management in WT. Its accurate interpretation directly informs risk stratification and provides the critical bridge between molecular alterations and current risk-adapted treatment protocols.

### 1.4. Current Treatment Protocols

Building on the epidemiologic, genetic, and histopathologic framework outlined above, modern management of WT exemplifies risk-adapted pediatric oncology. In high-income settings, multimodal therapy, consisting of surgery, chemotherapy, and selective radiotherapy, achieves overall survival exceeding 90%, with treatment tailored by stage, histology (favorable vs. anaplastic), and increasingly by molecular biomarkers [6,20].

Two cooperative groups anchor current practice: the COG in North America and the SIOP-Renal Tumour Study Group (SIOP-RTSG) in Europe and other regions. Although differing mainly in the timing of surgery and chemotherapy, both aim at maximizing the curative effect while minimizing acute and long-term toxicity [3,8].

COG protocols generally favor upfront radical nephrectomy for unilateral WT, followed by adjuvant therapy based on surgical–pathologic stage, histology, and key biologic markers, including loss of heterozygosity (LOH) at 1p/16q, 11p15 abnormalities, and emerging incorporation of 1q gain [3,8]. In contrast, SIOP-RTSG protocols typically administer preoperative chemotherapy (vincristine and actinomycin D ± doxorubicin for 4–6 weeks) to reduce tumor volume, limit intraoperative rupture, and facilitate nephron-sparing surgery, with postoperative therapy assigned according to post-chemotherapy histologic risk group and stage [14]. Both strategies achieve comparable outcomes for favorable-risk disease, with ongoing trials emphasizing harmonized endpoints and biomarker integration. Radical nephrectomy with regional lymph node sampling remains the cornerstone for unilateral resectable tumors, as omission risks understaging and undertreatment [3,6]. Nephron-sparing surgery (NSS) is increasingly employed for bilateral WT, patients with predisposition syndromes (e.g., *WT1*-related disorders, Beckwith–Wiedemann spectrum), solitary kidney, or selected unilateral tumors responding well to preoperative therapy [21].

WT is highly chemosensitive, with vincristine, actinomycin D, and doxorubicin forming the therapeutic backbone. Low- and standard-risk favorable-histology disease is treated with reduced-intensity regimens to preserve optimal survival while limiting anthracycline exposure and radiotherapy [14,20]. Higher-stage disease or adverse biomarkers (e.g., LOH 1p/16q, 1q gain, specific 11p15 contexts) prompt treatment intensification and selective radiotherapy, delivered with dose and field reduction to minimize late effects [6,8,20].

Diffuse anaplastic WT and post-chemotherapy blastemal-type WT remain challenging due to intrinsic chemoresistance and require aggressive multimodal intensification [14]. Approximately 15% of patients relapse; salvage strategies combine surgery, platinum/ifosfamide-based chemotherapy, and selected re-irradiation, with outcomes largely determined by prior therapy and relapse patterns [22]. High-dose chemotherapy with stem-cell rescue, targeted agents, and immunotherapy remain investigational.

Parallel efforts focus on integrating broader molecular profiling and exploring precision strategies, such as IGF axis inhibition, WNT/β-catenin modulation, and epigenetic therapies [8].

The immune system plays a crucial role both in the control of tumors and in cancer development and progression. On one hand, effector mechanisms of the innate and adaptive immune response contribute to the recognition and elimination of tumor cells; on the other hand, through the immunoediting process, the immune system itself is responsible for the selection of tumor variants able to escape from recognition, thus facilitating cancer progression. In addition, for many solid tumors, it has been clearly demonstrated that the TME can deeply influence the course of the disease, not only by favoring tumor survival and progression but also by shaping the immune cell populations infiltrating the tumor [23,24,25]. In the case of WT, characterized by a peculiar triphasic histology, the situation is even more complex, since each tumor component (epithelial, stromal, and blastemal) displays distinct features and could be variably susceptible to the immune response [26,27,28].

## 2. The Inflammatory Microenvironment in WT

The importance of the inflammatory microenvironment in driving and shaping immune cell response and events of tumor progression such as epithelial–mesenchymal transition (EMT) has been extensively reported in adult tumors [29,30,31,32]. Although less information is available regarding pediatric cancers, especially for relatively rare tumors like WT, more recent studies performed using omics approaches and based on the analysis of molecular databases have enabled the identification of inflammatory/immunological subtypes characterizing the immunological landscape of these tumors and correlating them with disease prognosis and outcome.

### 2.1. Leukocyte Populations Infiltrating WT

In WT, an inflammatory microenvironment characterized by the presence of inflammatory markers such as cyclooxygenase-2 (COX-2), hypoxia-inducible factor 1α (HIF-1α) vascular endothelial growth factor (VEGF), phosphorylated STAT3, phosphorylated extracellular signal-related kinases 1 and 2, inducible nitric oxide synthase (iNOS), and nitrotyrosine has been described [33,34,35]. Moreover, the immunohistochemical analysis of 14 WT tissue samples belonging to different stages (I–IV) collected at the time of diagnosis revealed the prevalent localization of these inflammatory molecules in the stromal component of the tumor and, to a lesser extent, in the blastemal and epithelial areas [35]. Importantly, the expression of inflammatory markers correlated with the presence of an immune cell infiltrate that included cells of both innate and adaptive immunity. In particular, tumor-associated macrophages (TAMs) represented the predominant cell type, and were primarily detected in stromal areas. Additionally, neutrophils and mast cells were found in almost all WTs analyzed, with similar preferential spatial distribution [36,37,38]. The presence of TAMs has been correlated to the clinicopathological features of the tumor. Indeed, CD68+ cell counts increased in stage IV compared with stage I [36], and TAM numbers in stage II WT were positively correlated with decreased disease-free survival [37]. Investigation of macrophage polarization in tumor tissues has revealed a prevalent anti-inflammatory M2 phenotype. Indeed, immunohistochemical and Western blot analyses, performed on 61 WT specimens, demonstrated higher positivity for M2 markers, including Arg-1, CD163, and CD206, compared to M1 proteins such as CD80 and iNOS, which were predominantly localized in the stromal component. In addition, the numbers of M1 TAMs decreased while those of M2 TAMs increased with increasing tumor stage (from stage I to stage III), suggesting a correlation between the M2 phenotype and poor prognosis in WT [38]. Interestingly, a recent study investigating macrophage heterogeneity in the WT TME identified BCL2A1 as a marker defining a subpopulation of tissue-resident macrophages associated with advanced tumors and poor prognosis [39].

Regarding adaptive immune cells, T lymphocytes were present in all tumor tissues, while B lymphocytes were present in 50% of samples analyzed. Both populations resided almost exclusively in the stroma. Although a low number of cases were studied, no differences in immune cell infiltration or the expression patterns of inflammatory markers were observed among different stages of WT. Additionally, the levels of COX-2 expression and other inflammatory factors paralleled the extent of TAM infiltration in the stromal component of WT. In a mouse model of WT, generated via *WT1* ablation and *IGF2* upregulation, similar data were obtained. Moreover, it was shown that other immune cells, such as pDC and Treg lymphocytes, were recruited to the TME as a consequence of the production of chemokines specific for CCR5 and CXCR4 receptors; furthermore, the immunoregulatory cytokines IL-10 and TGF-β were upregulated [40]. These data support the idea that an immunosuppressive microenvironment can be present in WT.

In another study comparing the immune cell infiltrate in WT with PB immune cells from the same patient through cytofluorimetric analysis, it was shown that CD45+ leukocytes in the tumor tissue displayed higher proportions of macrophages and NK cells compared to those in PB. Moreover, both CD4+ and CD8+ T cells were enriched in the effector memory subset and expressed CD57, HLA-DR, and PD-1 activation markers [41]. Notably, the composition of the immune cell infiltrate observed did not vary depending on timing of chemotherapy, as some patients had received chemotherapy before tumor resection, while others did not.

Recently, immunogenomic and proteomic studies based on omics techniques and public databases providing transcriptomic data (e.g., TARGET) have enabled the analysis of a large number of WT samples, leading to the identification of molecular immunological subgroups that correlated with prognosis and overall survival. A systematic analysis performed on public RNAseq data (TARGET) from 118 WT specimens and other pediatric solid tumors, including neuroblastoma (NBL), osteosarcoma (OS), clear-cell sarcoma of the kidney, and renal rhabdoid tumor, evaluated the Immune Constant of Rejection (ICR) score, which is related to the expression of 20 genes that reflect an active Th1/cytotoxic immune response [42]. ICR scores measured for all samples were correlated with survival rates. In WT, low ICRs were generally found and, in contrast to other tumors such as OS and high-risk NBL, lower ICR values corresponding to low immune infiltration were associated with better survival. This reverse correlation has also been described in clear cell renal cell carcinoma in adult patients [43]. Similar information was obtained when single-sample gene set enrichment analysis (ssGSEA) was performed to identify immune signatures characterizing the TME. Six different immune subtypes were identified: S1 (Th2-dominant), S2 (inflammatory), S3 (immunologically quiet), S4 (wound-healing), S5 (macrophage-dominant), and S6 (immune/lymphocyte-suppressed). Most WT samples belonged to the S3 and, at a lower proportion, to the S4 clusters. These subtypes were then correlated to overall survival. As shown for ICRs, the inflammatory S2 group in WT, characterized by high Th1 and low Th2 infiltration, was associated with worse prognosis; meanwhile, for other tumors, it was significantly correlated with better overall survival. However, the number of WT samples belonging to the S2 subtype was very small and did not allow for statistical analysis, while this could be performed between the S3 and S4 groups. In this case, WT samples with the S3 signature showed a better overall survival than those of the S4 subtype. The “immunologically quiet” S3 WTs were characterized by a low ICR score and low immune cell infiltration, with decreased numbers of Th1 and Th17 cells and high TGF-β expression. The “wound-healing” S4 WT tissues displayed higher infiltration compared to S3 tumors; however, this infiltrate was mainly composed of immune cells associated with an immunosuppressed microenvironment, such as Th2 and Treg cells and M2 macrophages. Moreover, the increased expression of wound-healing genes characterizing the S4 subtype, along with the IDO1 and LAG3 immunoregulatory molecules, further supports the correlation between an immunosuppressed TME and worse overall survival. From a therapeutic point of view, these results suggest that a different immunotherapeutic approach should be used for S3 and S4 WT. Indeed, the use of ICI would not be efficacious for the treatment of S3 tumors, in which low infiltration is present; in this context, the employment of tumor-targeting adoptively transferred immune cells would represent a better option. In contrast, for WT showing an S4 signature, targeted therapies aimed at disrupting immunosuppressive mechanisms would be of benefit.

In another study, immune-related gene signatures (IRCs) based on the ImmPort database were investigated in 122 WT cases, in order to identify possible different patterns of immune status and immune cell infiltration and to associate them with different disease outcomes. The aim of the study was the identification of prognostic markers reflecting immunologic status in the TME and predicting response to immunotherapy [44]. This analysis led to the identification and validation of 117 differentially expressed IRGs in WT specimens compared to adjacent normal tissue that reflected the landscape of immune infiltrate and status in the TME. Among these IRGs, 12 could be clustered into immune-related signatures reflecting immunophenotypic heterogeneity and showed prognostic value in predicting patients’ survival.

Very recently, another study addressed the same issue through integrative proteogenomic characterization of 91 WT samples compared to normal tumor-adjacent tissues [45]. The study combined proteomics and phosphorylated proteomics with whole-exome sequencing and transcriptome analysis, with the aim of identifying molecular markers and therapeutic targets to define additional clinical risk parameters for use in therapy selection. The obtained data revealed the existence of three distinct subgroups, each showing particular clinical, pathological, and molecular signatures. Each subgroup corresponded to one of the histological components: S1—blastemal, S2—stromal, S3—epithelial dominant. In line with the predominant blastemal component, the S1 subgroup was associated with poor outcome. Importantly, regarding the immunological aspects, S3 showed the highest immune score, with high expression levels of immune cell markers, HLA, and interferon-induced proteins. Moreover, it had a larger immune cell infiltrate than the S1 and S2 subgroups, enriched in M2 macrophages, γ/δ T cells, mast cells, CD8, and resting CD4 memory T lymphocytes, and displayed higher expression of chemokines, cytokines, and interferons. Notably, the S3 subgroup also presented significantly high expression of immune checkpoint molecules, such as CD274, VTCN1, and BTN3A1, suggesting the presence of an immunosuppressed TME. These results highlight the possibility of classifying WT patients according to proteogenomic signatures, supporting further studies investigating precision therapies specifically designed for a particular subgroup. In this context, targeted immunotherapies aimed at disrupting immune evasion mechanisms could be effectively applied for the treatment of WT belonging to the S3 type.

Recently, multi-omics analyses have also demonstrated how metabolic and mitochondrial-related gene signatures can influence immune infiltration, molecular subtypes, and drug sensitivity in adult renal malignancies. In clear cell renal cell carcinoma (ccRCC), the analysis of nuclear mitochondria-related genes (nMTRGs) revealed 11 genes differentially expressed in 530 ccRCC samples, with better prognosis in patients showing high expression of these genes [46]. Importantly, tumors showing low nMTRG expression displayed higher numbers of infiltrating NK cells, macrophages, and B lymphocytes; lower infiltration of CD4+ and CD8+ T cells; and high levels of CTLA4 and LAG3 expression, when compared to high-nMTRG tumors. The latter, in contrast, displayed higher CD274 (PD-L1) and HAVCR2 (TIM-3) levels. Further analysis predicted a lower ICB efficacy for the low-MTRG group. These findings further support the relevance of novel integrated immunogenomic approaches that could support tumor characterization and related therapeutic interventions, including in pediatric tumors such as WT.

### 2.2. NK Cells in WT

NK cells are a lymphocyte population of innate immunity that play an important role in antitumor immune response by exerting potent cytotoxic activity and producing cytokines, such as IFN-γ and TNF-α, upon interaction with cancer cells [47]. The study by Holl et al. was the first to describe the presence of NK cells in the WT microenvironment. Notably, these cells were increased in tumor specimens compared to PB, indicating a possible role of these innate lymphocytes in antitumor response against WT [41]. The presence of NK cells was further confirmed by immunohistochemical analysis of tissue sections from 19 WT specimens, which showed a prevalent localization of NKp46+ cells within the blastemal and epithelial components [27]. Although no additional information is available so far about the recruitment of NK cells in WT tissues and the possible presence of defined functional NK cell subsets, it is conceivable that these cells of the innate immune system may play a role in the antitumor response against WT. Indeed, in vitro studies aimed at characterizing the molecular and functional interactions between NK lymphocytes and WT cells have recently reported different outcomes depending on the activation state of the NK cells and on the cytokine milieu conditioning the phenotype and activity of immune and tumor cells [27,28].

## 3. Mechanisms of Tumor Escape from the Immune Response in WT

**Figure 2:**
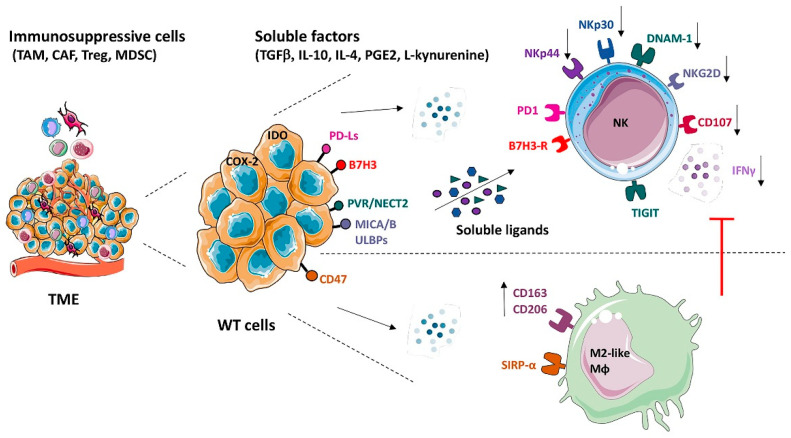
Cellular and molecular interactions occurring in the TME of WT and mechanisms of tumor escape from immune response. In the TME, different cell types are present that support tumor growth and progression, including vascular endothelial cells, tumor-associated fibroblasts (TAFs), and immune cells. WT cells produce various soluble factors, constitutively or induced by the conditioning milieu, and express inhibitory surface molecules that shape immune response toward an immunosuppressed state. NK cells and macrophages are reported targets of tumor-mediated inhibition. NK cell activation and function is impaired by soluble mediators—including regulatory cytokines, IDO, PGE2, and soluble ligands for activating receptors—and through the engagement of inhibitory surface receptors such as B7-H3, PD-1, and TIGIT. M2 polarization of macrophages is promoted through mechanisms possibly involving soluble mediators such as PGE2, released by tumor cells upon physical interaction with phagocytes. The figure contains modified images from Servier Medical Art (https://smart.servier.com, accessed on 12 January 2026), licensed by a Creative Commons Attribution CC BY 4.0 International License (https://creativecommons.org/licenses/by/4.0/).

Different effector cells of both innate and adaptive immunity are recruited to the tumor site to exert the antitumor response. However, these cells may display functional alterations due to the capability of tumor cells to inhibit an effective immune response. This may be due to different tumor-developed strategies involving the production of soluble inhibitory factors and cell-to-cell contact interactions [48,49,50,51]. Regarding WT, the cellular and molecular interactions taking place in the tumor microenvironment between WT and immune cells have only partially been investigated. Moreover, in this tumor, it must be considered that the different histologic components may exert different effects on immune cells, depending on component-specific phenotypic and functional characteristics. In this context, stromal-type WT cells have been shown to display morphological, phenotypic, and biological features typical of mesenchymal stem cells (MSCs) [52]—a cell type known to display a potent immunosuppressive effect on cells of both innate and adaptive immune systems, including NK cells and macrophages [53,54,55,56,57]. It has been reported that WT putative cancer stem cells (CSCs), identified as CD56+CD133−ALDH1^bright^ cells, reside in the blastemal component [26]. This cellular subset may also display unique properties in conditioning the immune response in the TME of WT. Figure 2 summarizes the main evasion mechanisms acting in the TME of WT, with a particular focus on the interactions established by WT cells with NK lymphocytes and macrophages.

In the TME, the production of inhibitory factors, including IDO and PGE2, represents a widely shared mechanism of immunosuppression of antitumor response in many cancers [58,59,60,61]. In WT, both IDO and PGE2 are produced by tumor cells upon stimulation by IFN-γ and IL-1β+TNF-α, respectively, and are responsible for the inhibition of NK cell activation [28].

A major mechanism of immune evasion is the expression of inhibitory ligands by tumor cells, which are recognized by inhibitory receptors that negatively regulate the function of different cells in both innate and adaptive immunity [62,63,64,65,66]. WTs have been shown to express several immune checkpoint molecules, including PD-L1, B7-H3, VTCN1, CD47, and VISTA, with B7-H3 displaying the highest expression levels [28,42,45,67]. Other ligands that may exert an inhibitory effect on immune cells are represented by soluble HLA class I molecules. Soluble-HLA-G has been shown to trigger apoptosis in activated CD8+ T cells and suppress both NK and cytotoxic T lymphocyte responses [68]. To date, this possible mechanism of immune evasion has not been explored in WT.

Another mechanism of immune escape possibly acting in the TME involves tumor cells releasing soluble ligands for activating receptors. For example, the proteolytic shedding that generates soluble NKG2D ligands (sNKG2DLs) is involved in sNKG2DL-driven remodeling of the TME toward an immunosuppressive state; as such, it is now targeted in novel immunotherapeutic approaches [69,70,71]. Soluble ligands for other activating NK receptors—namely, DNAM-1 and NKp30—have been detected in cancer patients, correlating with disease progression [72,73,74].

In the last several years, it has become increasingly evident that tumor-derived extracellular vesicles (EVs) play an additional role in the development and progression of tumors by influencing immune cells, including NK cells [48,75,76,77,78]. This aspect has been very poorly investigated in WT. A recent study reported that plasma EVs from 14 WT patients expressed significantly increased levels of PD-L1 compared to healthy subjects. These PD-L1+ EVs could inhibit the activation of human CD8+ T cells, as evidenced by the downregulation of the CD69 activation marker and the inhibition of IFN-γ and TNF-α production [79].

### 3.1. Mechanisms Acting on NK Cells

Regarding NK cells, very little information is available about the outcome of their interaction with WT cells. Two recently published studies have focused on the characterization of primary WT cell populations isolated and expanded from tumor tissues derived from nephrectomized WT patients and on the effects that these cells could exert on NK lymphocytes [27,28]. In the first study, WT cell lines expressing a blastemal CD56+/CD133− or an epithelial CD56−/CD133+ phenotype were characterized via cytofluorimetric analysis. Both cell types expressed ligands recognized by activating receptors, such as CD112 (Nectin-2), CD155 (PVR), ligands of DNAM-1 activating receptors, and ULBP2/5/6 and MICA/B, recognized by NKG2D. Moreover, they also expressed the immune checkpoint ligand PD-L1 and HLA class I molecules, with the latter recognized by KIR and NKG2A inhibitory receptors [27]. In the second paper, published by our group, WT cell populations of stromal type (CD105+, CD90+, CD73+, vimentin^high^, and cytokeratin^neg/low^) were isolated and analyzed, which showed a similar phenotype and expressed both DNAM-1 and NKG2D ligands. Additionally, they stained positively for sNKp46, sNKp30, and sNKp44 soluble receptors, indicating that they expressed the putative ligands of Natural Cytotoxicity Receptors (NCRs). Regarding the ligands for inhibitory receptors, stromal WT cells expressed the inhibitory checkpoint molecules B7-H3, PD-L1/L2, CD47, and HLA class I molecules. Notably, B7-H3 was expressed at very high levels.

When NK cell populations from healthy donors previously activated with IL-2 were tested in cytotoxicity assays against WT cells, they could efficiently produce IFN-γ and TNF-α, degranulate, and lyse tumor cells independently of the target histologic phenotype [27,28]. These findings indicate that, when present in an active state, NK cells can recognize and kill WT cells as a result of predominant activation rather than inhibitory interactions. A completely different effect was observed when freshly isolated, resting NK cells were used in co-culture experiments with tumor cells. Under these conditions, NK-mediated WT cell lysis did not occur, while IL-2-induced activation of NK lymphocytes was inhibited by WT cells. In particular, NK cell proliferation was significantly impaired by stromal-type WT populations, and this inhibition was due to the production, by tumor cells, of PGE2 and IDO inhibitory factors. Inhibition affected also the acquisition of an activated phenotype, as co-cultured NK cells showed a lack of upregulation of NKp30, NKp44, NKG2D, and 2B4 activating receptors [28]. The immunosuppressive effect was mediated by inhibitory factors produced by stromal WT cells upon interaction with NK cells. IDO- and PGE2-mediated inhibition required cell-to-cell contact, as it was not observed in culture experiments performed under Transwell conditions.

Physical interaction caused the production of IFN-γ and TNF-α by NK cells, which induced COX-2 and IDO expression in WT cells, as well as the upregulation of inhibitory ligands, including HLA class I, PD-L1/L2, and CD47 molecules, thus promoting tumor immunosuppressive behavior.

In the study by Fiore et al., both the epithelial and blastemal components were studied and shown to impair NK cell-mediated cytotoxic activity against K562 erythroleukemia cells, reduce IFN-γ production, and inhibit the IL-2 mediated upregulation of NKp30, NKp44, DNAM-1, and TIM-3 receptors [27]. Additionally, in this case, impairment required cell-to-cell contact; however, it was not prevented by the addition of IDO and PGE2 inhibitors, thus excluding a possible involvement of these immunosuppressive factors.

In conclusion, the data published so far indicate that, depending on their activation state, NK cells may be susceptible to WT-mediated inhibition, while they could also kill tumor cells. The results of the interaction may depend also on the cytokine milieu of the tumor tissue, as cytokines such as IFN-γ, IL-1β, and TNF-α—which may be present in the TME—can induce the production of inhibitory factors in WT cells that are involved in inhibitory effects.

### 3.2. Mechanisms Acting on Macrophages

Macrophages appear to be the most prevalent immune cell type in WT tissues, with preferential localization in stromal areas. Different studies addressing the identification of immune signatures in WT specimens possibly related to disease prognosis and outcome have revealed that M2-polarized cells are the main macrophage type present in the TME. This evidence further supports the existence of an immunosuppressive microenvironment in WT that conditions the phenotype and function of recruited immune cells. However, little is known about the mechanisms involved in M2 polarization of macrophages by WT cells. A recent study reported that peripheral blood (PB)-derived monocytes differentiated into M0 macrophages in the presence of epithelial or blastemal WT cell populations and showed an M2-polarized phenotype, as indicated by higher expression of CD163 and CD206 M2 markers [27]. Moreover, these M2-like cells could in turn negatively regulate NK cell effector functions, as degranulation and autologous IFN-γ production upon contact with K562 target cells were decreased after prior co-culture of NK lymphocytes with M2-like macrophages. The polarizing effect of WT cells was contact-dependent, as it was not observed in Transwell experiments.

### 3.3. Mechanisms Acting on Other Immune Cells

Pediatric solid tumors, including WT, are typically characterized by a low mutational burden and limited neoantigen repertoire, factors that constrain T cell activation and partly explain the modest responses observed with immune checkpoint inhibitors in children. In this context, tumor-infiltrating T lymphocytes often display reduced effector function and may require cooperative activation by antigen-presenting cells, such as dendritic cells, to generate durable antitumor responses and immune memory [80]. In WT, different reports have described an immune infiltrate characterized by a low presence of T cells [35,40,81] in the tumor tissue. In most cases, T lymphocytes are mainly represented by Th2 and Treg cells, as confirmed through immunohistochemical, transcriptomic, and proteomic studies [40,42,45], correlating with an immunosuppressive microenvironment. In another study, both CD4+ and CD8+ T cells found in WT displayed an activated effector memory phenotype and, more importantly, expressed PD-1 inhibitory receptor, further supporting evidence of an inhibited state of immune cells in WT [41]. Moreover, tumor-infiltrating lymphocytes derived from pediatric tumors, including 5 WTs, were shown to be defective in terms of in vitro activation and expansion, when compared to normal control cells, as a consequence of the original conditioning exerted by the tumor involving the low expression of HLA class I, ICAM-1, and LFA3 molecules, as well as the significant production of TGF-β [81]. Apparently in contrast with these findings, a study reported that higher numbers of CD8+ T cells in WT tissues, localizing at the invasive margins, were positively correlated with better disease outcomes, particularly in early-stage WT [82]. However, it could be considered that, when the T-cell-mediated response is not counteracted by the tumor, it can play a positive and effective role in the antitumor immune response. Despite the availability of different studies performed directly on WT tissues, in vitro studies using tumor-derived cell populations and aimed at deeply investigating the molecular interplay and outcome of the interaction between T lymphocytes and WT cells—and, thus, possibly clarifying the active mechanisms in the TME—remain elusive.

B cells and other immune elements, including dendritic cells, are increasingly recognized as modulators of antitumor immunity and may influence immunoediting and antigen presentation. Very little information is available regarding both B lymphocytes and dendritic cells and their specific roles in WT. In the study by Maturu et al., B lymphocytes were found in 7 out of 14 WT tissues analyzed via immunohistochemistry, making them less frequent compared to T cells [35]. However, no additional informative data are available regarding the phenotype and function of B cells in the WT TME. Moreover, in tumor tissues of a WT mouse model, plasmacytoid dendritic cells were significantly present, correlating with the observed increased expression of IDO1 and supporting an immunosuppressive role of these cells [40].

Taken together, these observations highlight the need for a broader, integrated view of the WT immune landscape, in which innate and adaptive immune compartments interact dynamically, providing a rationale for combined immunotherapeutic approaches aimed at enhancing antigen presentation, T cell activation, and long-term immune surveillance. In this regard, the use of tumor-derived 3D-structure organoids in vitro or patient-derived xenograft (PDX) models in vivo could allow for deeper investigations, with tumor models maintaining the original cellular heterogeneity of WT (for WT organoids) and PDX models maintaining the other components of the TME.

## 4. Preclinical and In Vitro Models for WT

Recent advances in cancer research have highlighted the importance of integrating molecular profiling, immune landscape characterization, and clinically relevant preclinical models to improve therapeutic development. PDX models have emerged as valuable platforms that preserve tumor heterogeneity and microenvironmental features, enabling translational investigation of tumor progression and treatment response across multiple cancer types [83]. In the context of WT, the study by Murphy et al. demonstrated the relevance of PDX models as a tool to study the complexity and heterogeneity of WT by deriving xenografts from 45 WT patients, including those with diffuse anaplasia, disease relapse, or bilateral tumors. Interestingly, it was shown that WT-PDXs maintained the expected treatment resistance or sensitivity of their histological subtypes [84]. In parallel, other experimental tools are being developed, with the aim of capturing the heterogeneity of pediatric solid cancers, such as WT-derived three-dimensional (3D) spheroids [85] and WT-derived 3D organoid culture models [86]. Although not yet applied to WT, the technology of microfluidic cell culture represents a challenging yet promising tool to generate human organs-on-chips—a very useful preclinical model in cancer research enabling the study of crucial issues, including tumor cell behavior, tumor–TME interactions, and anti-cancer drug development [87].

## 5. Novel Therapeutic Approaches in WT

Conventional therapies for WT patients include surgery, pre- or postoperative chemotherapy and, in some cases, radiotherapy. Although these protocols are effective in most cases of WT with favorable histology, yielding a 5-year survival rate of about 90%, alternative therapeutic strategies are needed; this is especially true for patients with high-risk tumors, in which the efficacy of standard therapies is reduced and the possibility of recurrence is increased. In addition, the development of novel approaches to be used in combination with standard treatments could also be beneficial in reducing the adverse effects associated with chemo- and radiotherapy and the risk of long-term complications—a major concern, especially in pediatric patients.

### 5.1. Immunotherapy

Immunotherapy represents one of the main alternative strategies for cancer treatment, with the goal of targeting and eliminating tumor cells through the enhancement of the patient’s immune response. Several immune-based approaches have been developed for adult patients and have been shown to be effective in different hematological and solid malignancies. In this context, the targeting of immune checkpoints (ICs) and the engineering of immune cells with Chimeric Antigen Receptors (CAR) are currently among the most promising and investigated strategies [66,88]. The translation of immunotherapy to pediatric solid tumors is still challenging; while the issues related to the presence of the TME, possibly generating an immunosuppressive milieu, are shared with adult cancers, pediatric tumors are generally characterized by a lower mutational burden and are poorly immunogenic [89,90]. For WT in particular, an additional obstacle is represented by its rare nature and the low number of patients that are recruited in broad clinical trials aimed at evaluating novel immunotherapeutic approaches for different types of pediatric solid tumors (Table 1).

In view of the above considerations, the identification of molecules highly expressed in WT is crucial for the design of immune-based treatments capable of selectively targeting tumor cells. Among the most studied targets, ICs include several surface molecules which are able to interact with receptors expressed by different immune cells and impair their functional properties [65,66,91]. The inhibition of IC pathways represents an effective strategy for cancer treatment. In particular, the use of monoclonal antibodies (mAbs) blocking the interaction of PD-1 or CTLA-4 with their corresponding ligands (PD-L1/L2 and CD80/CD86, respectively) has shown remarkable efficacy in different solid tumors, including melanoma and lung carcinoma [62,63]. Consequently, the same strategy is currently being explored for pediatric solid cancers [65,92]. The PD-L1 molecule has been extensively analyzed in several pediatric tumor types, revealing quite low expression, with the exception of Hodgkin’s lymphoma and sarcoma [92,93,94,95,96]. Several studies focused on WT have confirmed that PD-L1 is generally expressed at low levels and in a small percentage of patients, although heterogeneous results have been obtained. Interestingly, PD-L1 expression is more frequent in anaplastic WT and correlates with disease recurrence. A similar association has also been reported in WT with favorable histology, independent of tumor stage [97,98]. In recent years, some WT patients have been included in clinical trials based on IC inhibitors (Table 1). Two trials have used atezolizumab or pembrolizumab to block the PD-1/PD-L1 axis (NCT02541604; NCT02332668) [99,100], while another one (NCT01445379) included ipilimumab, an anti-CTLA-4 mAb [101]. Although well tolerated, the overall efficacy of these therapies in WT patients was poor. In the phase I-II KEYNOTE-051 study (NCT02332668), pembrolizumab showed an acceptable safety profile in children with advanced cancers but produced objective responses in only a small proportion of Hodgkin pediatric lymphomas, underscoring the limited efficacy of single-agent checkpoint inhibition in this setting [100]. Similarly, the phase I-II iMATRIX study (NCT02541604) showed that atezolizumab was well tolerated but displayed limited efficacy [99]. Additionally, the NCT0101445379 study, based on the targeting of CTL4 through ipilimumab, showed no objective tumor regressions [101].

In addition to PD-1 and CTLA4, other ICs have been investigated in WT, including HVEM, Gal-9, and MHC class II, which bind BTLA, TIM-3, and LAG-3, respectively. Although the number of samples analyzed was too low to draw definite conclusions, none of these molecules appear to be homogeneously expressed in a substantial proportion of WT [65,92]. It is notable that, while immune checkpoint inhibitors have demonstrated unprecedented efficacy in several adult malignancies, they have shown modest activity in most pediatric cancers, likely reflecting their generally low mutational burden and limited neoantigen landscape, which contribute to reduced intrinsic immunogenicity and diminished responsiveness to checkpoint blockade [80]. Consequently, increasing attention has shifted toward therapies directed against tumor-associated surface antigens. Among these, the B7-H3 (CD276) surface molecule has emerged as a promising target, given its widespread expression in pediatric solid cancers and its role as an immune inhibitory ligand. B7-H3, a checkpoint member of the B7 family [102,103], is broadly expressed by solid tumors of various histotypes [104,105]. Initially characterized as an excellent biomarker and target in neuroblastoma (NB), it was subsequently found to be highly expressed in different pediatric solid cancers, including WT [65,67,105]. B7-H3 acts as an immune checkpoint ligand; although the surface molecule recognized by B7-H3 on T and NK cells is still unknown, the engagement of its receptor results in the inhibition of T- and NK-cell-mediated effector functions against tumors [102,106]. Notably, B7-H3 expression correlates with poor prognosis, resistance to therapy, and increased metastasis in several cancer types [91,107]. Humanized affinity-matured anti-B7-H3 antibodies have demonstrated potent antitumor activity and the capacity to modulate immune inhibition in preclinical models, supporting their investigation as potential therapeutic tools in WT and other embryonal malignancies [108]. Based on these findings, the mAb-mediated targeting of B7-H3 has been the focus of several clinical trials concerning pediatric tumors [67,91]; the NCT02982941 trial is based on enoblituzumab (MGA271) and also includes WT patients, but the results are not yet available (Table 1). Drug-conjugated anti-B7-H3 mAbs are also under investigation, and their efficacy has been established in in vitro and in vivo preclinical models. The m276-SL-PBD mAb displayed antitumor activity against WT patient-derived and cell line-derived xenografts [109], while votramitamab duocarmazine (MGC018) efficacy was assessed in preclinical models of pediatric sarcoma and NB [110,111,112]. Another strategy to target B7-H3-expressing tumors is the design of B7-H3 Chimeric Antigen Receptor (CAR)-T cells [67,113,114]. Several clinical trials are ongoing in pediatric cancer patients, two of them (NCT04483778 and NCT04897321) also enrolling children affected by WT (Table 1). In particular, NCT04483778 (STRIvE-02)—a phase I study in patients with relapsed/refractory solid tumors—showed that B7-H3 CAR-T cells are tolerable and display limited antitumor activity, without causing acute on-target, off-tumor toxicity [115].

In addition to ICs, other surface molecules expressed by WT may represent attractive targets for immunotherapy, especially if highly expressed by tumor cells and in low amounts in normal tissues. Carcinoembryonic antigen glypican-3 (GPC3) is a heparan sulfate proteoglycan involved in cell growth and development which is overexpressed in primary and metastatic WT [116,117,118]. Like B7-H3, possible therapies targeting GPC3 comprise not only mAbs but also GPC3-specific CAR T cells [119,120]. Both approaches are being evaluated in different clinical trials in which WT patients are enrolled. In one trial (NCT04928677), the therapeutic agent is codrituzumab, while others (NCT05103631, NCT04377932, and NCT04715191) are utilizing GPC3-specific CAR-T cells engineered with different constructs (Table 1). A phase I study of GPC3-derived peptide vaccine therapy for patients with refractory pediatric solid tumors, including one WT patient, showed a good safety profile and the ability to induce a GPC3-specific immune response. Two different GPC3 peptides were used, restricted for HLA-A*24:02 and for HLA-A*02:01, respectively. It was shown that GPC3 peptide-specific CTLs could recognize GPC3+ tumor cell lines and were able to infiltrate tumor tissues. Moreover, GPC3-specific CTL frequency was correlated with progression-free survival and overall survival in patients vaccinated with the GPC3 peptide [121].

In the search for additional targetable WT markers, several studies have focused on gangliosides since it has been shown that the differential glycosylation of glycoproteins or their aberrant expression is a frequent hallmark of cancer cells [122]. NeuGcGM3 (N-glycolylated ganglioside monosialic 3) is a glycosphingolipid expressed on the surface of various tumors, including all WT subtypes, but absent in healthy tissues [123,124]. A phase I clinical trial (NCT01598454) evaluated an anti-idiotype vaccine targeting NeuGcGM3 (racotumomab) in children with relapsed or resistant tumors, showing a favorable toxicity profile and immunogenicity. This vaccine elicited an immune response in most patients, as measured via the detection of anti-NeuGc-GM3 antibodies, but it was not effective in the single WT patient enrolled in the study. Although racotumomab did not display significant antitumor activity in most patients, its low toxicity combined with its immunogenicity is promising for the future investigation of long-term vaccination protocols [125].

Among other therapeutic targets that are currently being explored for pediatric solid tumors, the CD56 molecule displays a broad expression in all WT histological types but is enriched in the blastemal component [26,126]. The targeting of CD56^+^ pediatric tumors has been evaluated in a phase II study (NCT02452554) (Table 1) through the use of an antibody–drug conjugate (lorvotuzumab mertansine), consisting of a potent antimitotic (DM1) linked to a CD56-targeting antibody. Although preclinical data demonstrated the in vivo effects of lorvotuzumab mertansine against WT and other pediatric tumors [127], this strategy showed limited efficacy in the 17 enrolled children with relapsed or refractory WT [128].

The adoptive transfer of antigen-specific T cells is a possible strategy to target tumor-associated antigens (TAAs) and redirect the immune response against cancer. The targeting of multiple antigens could represent a good strategy to counteract antigen loss—a common mechanism exploited by tumors to evade the immune response. Two phase I clinical trials (NCT02789228, NCT05238792) are investigating the administration of multiTAA-T products consisting of autologous T cells expanded ex vivo through stimulation with peptide-pulsed antigen presenting cells. The NCT02789228 trial enrolled pediatric and adult patients with relapsed/refractory solid tumors (including 7 WT cases), where the targeted TAAs were Wilms tumor gene 1 (WT1), preferentially expressed antigen of melanoma (PRAME), and survivin [52,129,130,131]. Encouraging results have been reported, showing that TAA-T administration is safe and can prolong the time to progression [132].

Along with therapeutic options aimed at directly targeting tumor cells, researchers are also focusing on approaches to counteract different immune suppressive mechanisms acting in the inflammatory TME. For example, blockade of COX-2 or IDO through specific inhibitors has been shown to impair tumor growth and progression in in vivo preclinical models [133]. COX-2 expression is generally high in WT and associated with immune suppressive properties and worse prognosis [27,28,34,40,134,135]. Several studies have investigated the use of COX-2 inhibitors in combination with other therapeutic agents, as in the case of the NCT02574728 trial, in which patients with different tumor types were enrolled and celecoxib was used (Table 1). Additionally, IDO inhibitors are currently under investigation; for example, indoximod in combination with chemotherapy is being assessed in clinical trials involving pediatric malignant brain tumors [136], but no studies involving WT patients have been reported to date. The targeting of angiogenesis represents an alternative option to modulate the TME and indirectly target the tumor [137]. In the case of WT, strategies focused on the VEGF/VEGFR pathway have utilized anti-VEGF mAbs or VEGFR-directed kinase inhibitors in combination with chemotherapy [138,139,140].

Overall, the clinical experience with immunotherapy in WT remains preliminary and largely derived from early-phase, basket, or multi-histology trials enrolling small numbers of WT patients. Immune checkpoint inhibitors targeting the PD-1/PD-L1 and CTLA-4 axes have shown limited efficacy in unselected WT populations, likely reflecting the low tumor mutational burden and the immunosuppressive, stromal-rich microenvironment characteristic of this tumor. In contrast, strategies directed against highly expressed surface molecules such as B7-H3, as well as multi-tumor-associated antigen adoptive T-cell approaches, provide a stronger biological rationale, although definitive efficacy data are still pending. Importantly, for many of the trials listed, conclusive and WT-specific results have not yet been fully published, limiting comparative assessment. These considerations underscore the need for biomarker-driven patient selection and rational combination strategies aimed at overcoming the immune resistance mechanisms intrinsic to WT.

The clinical trials summarized in Table 1 illustrate both the growing interest in the application of immunotherapeutic protocols for WT and the challenges that have thus far limited its clinical impact. Despite the inclusion of WT patients in several early-phase studies, the overall evidence indicates that immunotherapy for WT is still exploratory, with heterogeneous strategies, small numbers of patients, and largely preliminary outcomes.

### 5.2. Targeted Therapy

Increasing evidence supports the rationale for combining targeted pathway inhibitors with immunotherapeutic approaches to overcome the immunologically “cold” and immunosuppressive microenvironment typical of WT. Tumor-intrinsic activation of developmental pathways frequently dysregulated in WT, including Wnt/β-catenin and IGF signaling, can promote immune exclusion, stromal remodeling, and resistance to T-cell-mediated cytotoxicity. In different cancer types, activation of Wnt/β-catenin signaling has been consistently associated with non-T cell-inflamed tumor phenotypes and impaired dendritic cell recruitment, ultimately limiting T cell priming and responsiveness to ICIs. Conversely, pharmacologic inhibition of this pathway may restore immune infiltration and sensitize tumors to immunotherapy [141]. Regarding WT, it has been recently shown that, in WT specimens, higher levels of PD-L1 correlated with *CTNNB1* mutations, while activation of Wnt/β-catenin signaling increased PD-L1 expression in vitro [142], suggesting that blockade of this pathway could positively influence the response to ICI therapy.

Several targeted therapies do not directly modulate the immune response but, rather, aim to inhibit molecular pathways crucial for tumor development and progression [140] (Table 1). Examples of such strategies applied to WT include EGFR-specific CAR-T cells [143] (NCT03618381); disruption of the Wnt/beta-catenin pathway through inhibitors such as Tegavivint [144,145] (NCT04851119); inhibition of the PI3K/AKT pathway [140,146]; blockade of the IGF-2 signaling pathway [147]; and targeting of exportin-1 (XPO1) through selective inhibitors such as Selinexor [148] (NCT05985161). Notably, the use of IGF-2 inhibitors not only interferes with the IGF-2/IGF1R pathway, but can also exert the additional effect of inhibiting COX-2 expression and subsequent PGE2 synthesis, as COX-2 can be induced by IGF-2 signaling [40]. The rationale for XPO1 targeting derives from the finding that most WTs (and malignant rhabdoid tumors) display an aberrant activation of the nuclear export protein XPO1. Importantly, selixenor has been shown to induce cell cycle arrest and apoptosis in vitro, while it inhibited tumor growth in in vivo PDX models [149]. Moreover, an early-phase pediatric clinical trial (NCT02323880) using selinexor in patients with recurrent/refractory CNS and solid tumors demonstrated selinexor-related hematologic and neurologic toxicities, highlighting the need for dose reduction. In this context, the availability of next-generation XPO1 inhibitors with reduced CNS penetration could improve tolerability [150]. Regarding the Wnt pathway, Frizzled 7 (FZD7) plays a role in cancer promotion and progression through activation of the classical Wnt pathway and is overexpressed in blastemal WT [151,152,153]. Notably, a recent study described the generation of a novel anti-FZD7 mAb that blocks the Wnt pathway in WT and decreases the expression of CSC-associated markers, holding promise for its possible clinical translation [154].

Epigenetic alterations are frequently observed in different pediatric cancers [155]. Concerning WT, epigenetic dysregulation contributes to the impairment of normal kidney development, thus playing an important role in tumorigenesis [8,156,157,158]. In particular, changes in DNA methylation, histone modifications, and long non-coding RNA have been described [159,160,161]. As epigenetic modifications are reversible, the possibility of therapeutic targeting through specific modulators appears to be feasible and attractive. Promising candidates under investigation include inhibitors of histone deacetylases (HDACs) and Enhancer of Zeste Homolog 2 (EZH2)—chromatin-modifying enzymes that display an aberrant activity in WT cells and contribute to the maintenance of an undifferentiated tumor state, especially in the aggressive blastemal subtype [162,163]. Interestingly, in lung cancer, epigenetic therapies combining DNA methyltransferase and histone deacetylase inhibitors have demonstrated the ability to reverse immune evasion by enhancing interferon signaling, antigen presentation, and the expression of T cell-attracting chemokines, thereby creating a TME which is more permissive of immune-mediated clearance [164]. Therefore, the use of epigenetic modulators in combination with immunotherapeutic strategies could help to reprogram the WT microenvironment, enhance immune infiltration and activation, and ultimately improve the therapeutic response. Alterations in miRNA expression profiles have also been described in WT, which are being investigated both as possible biomarkers and as targets for innovative therapeutic approaches [8,157,159].

## 6. Conclusions

WT exemplifies how developmental biology, tumor histopathology, and immune regulation intersect to shape therapeutic vulnerability. Emerging evidence indicates that the immune escape mechanisms in WT are closely linked to its triphasic architecture and stromal-driven immunosuppressive microenvironment, providing a biological explanation for the limited efficacy of current immunotherapies in unselected patients. Translational strategies that are capable of overcoming the immunologically “cold” and heterogeneous TME may benefit from integrated molecular and immune profiling to identify biologically distinct subgroups and enable biomarker-driven patient stratification. Thus, tumors characterized by poor immune infiltration but which express targetable markers may be treated with therapies employing tumor-specific, adoptively transferred immune cells, such as B7-H3- and GPC3-CAR-T cells or multiTAA-T lymphocytes. On the other hand, for WT showing an immunosuppressive TME with significant expression of inhibitory checkpoint molecules, additional interventions aimed at disrupting inhibitory interactions may elicit proper activation and function of antitumor immune response. In this context, therapies combining immune checkpoint blockade and inhibition of IDO or COX-2 enzymes could represent an even more potent option. Finally, immunotherapy approaches may take advantage of the wide spectrum of effects exerted by some combined targeted treatments aimed at interfering with pathways supporting tumor survival and progression. For example, IGF-2 inhibitors—primarily designed to inhibit upregulated IGF-2 signaling—could also affect PGE2 production, as IGF-2 is involved in the induction of COX-2 expression. Taken together, these observations underline the need for prospective clinical trials incorporating comprehensive immune profiling—including spatial and single-cell characterization of tumor-infiltrating immune populations—to identify effective patient-specific therapeutic strategies. For this purpose, advancing immunotherapy in WT will require close collaboration among pathologists, immunologists, and clinicians to translate mechanistic insights into precise, less-toxic treatments for children with high-risk disease.

## Figures and Tables

**Table 1:** Immunotherapy and Targeted Therapy Clinical Trials Including Patients with Wilms Tumor.

Clinical Trial	Therapeutic Agent/Product	Class	Molecular Target/Pathway	Phase/Design	WT Inclusion	WT Patients (n)	WT-Specific Biological Rationale	Status
NCT02541604	Atezolizumab	mAb (immune checkpoint inhibitor)	PD-L1	Phase I/II, basket	Explicit	10	Variable PD-L1 expression in relapsed/anaplastic WT	Terminated, with results
NCT02332668	Pembrolizumab	mAb (immune checkpoint inhibitor)	PD-1	Phase I/II pediatric	Included	3	Safety/efficacy of PD-1 blockade in refractory WT	Recruiting
NCT01445379	Ipilimumab	mAb (immune checkpoint inhibitor)	CTLA-4	Phase I pediatric	Explicit	3	Enhancement of T-cell activation in immunologically cold WT	Completed
NCT02982941	Enoblituzumab (MGA271)	mAb	B7-H3	Phase I pediatric	Potential	n.r.	B7-H3 expression linked to aggressive WT	Completed
NCT05293496	Vobramitamab duocarmazine	ADC	B7-H3	Phase I	Not specified	n.r.	Targeted cytotoxic delivery via B7-H3	Completed
NCT04897321	B7-H3 CAR-T	CAR-T cell therapy	B7-H3	Phase I pediatric	Potential	n.r	High B7-H3 expression in WT	Recruiting
NCT04483778	B7-H3 CAR-T	CAR-T cell therapy	B7-H3	Phase I pediatric	Potential	n.r	Limited normal tissue expression of B7-H3	Active, not recruiting
NCT04928677	Codrituzumab	mAb	GPC3	Phase I/II pediatric	Potential	n.r.	GPC3 expression in WT subsets	Recruiting
NCT04377932	IL-15 armored GPC3 CAR-T	CAR-T cell therapy	GPC3	Phase I pediatric	Potential	n.r	IL-15 improves CAR-T persistence; GPC3 in WT	Active, not recruiting
NCT05103631	IL-15 armored GPC3 CAR-T	CAR-T cell therapy	GPC3	Phase I	Potential	n.r	Extension of armored CAR-T to WT	Recruiting
NCT04715191	GPC3 CAR-T (IL-15 and -21 armored)	CAR-T cell therapy	GPC3	Phase I pediatric	Potential	n.r	Dual cytokine armoring to overcome WT TME	Recruiting
NCT01598454	NeuGcGM3 mimic vaccine (Racotumomab)	Therapeutic vaccine	NeuGcGM3	Phase I pediatric	Included	1	Ganglioside expression in pediatric tumors incl. WT	Completed, with results
NCT02452554	Lorvotuzumab mertansine (IMGN901)	ADC	NCAM (CD56)	Phase II	Included	17	NCAM expression in WT	Completed, with results
NCT02789228	MTAA-Ts (multi tumor-associated antigen-specific T cells)	Adoptive T-cell therapy	Multiple TAAs	Phase I	Explicit	9	Multi-antigen targeting limits escape in WT	Active, not recruiting
NCT05238792	MTAA-Ts (multi tumor-associated antigen-specific T cells)	Adoptive T-cell therapy	Multiple TAAs	Phase I	Potential	n.r	Expanded multi-antigen strategy for WT	Recruiting
NCT02574728	Celecoxib (COX-2 inhibitor) (combination with sirolimus, etoposide, cyclophosphamide)	Repurposed combination	mTOR, COX-2	Phase II	Not specified	n.r	Targeting growth/angiogenesis pathways in WT	Active, not recruiting
NCT03618381	EGFR CAR-T	CAR-T cell therapy	EGFR	Phase I pediatric/AYA	Potential	n.r	EGFR expression in WT subsets	Active, not recruiting
NCT04851119	Tegavivint (TLB1 inhibitor)	Targeted small molecule	TBL1/Wnt–β-catenin	Phase I/II	Explicit	n.r	Developmental signaling dysregulation in WT	Recruiting
NCT05985161	Selinexor (XPO1 inhibitor)	Targeted small molecule	XPO1	Phase II	Explicit	n.r	Disruption of oncogenic nuclear export in WT	Recruiting

(Updated to January 2026) Abbreviations: WT, Wilms tumor; mAb, monoclonal antibody; n.r., not reported; ADC, antibody-drug conjugate; CAR, chimeric antigen receptor; NeuGcGM3, N-glycolylated ganglioside monosialic 3; NCAM, neural cell adhesion molecule; EGFR, epithelial growth factor receptor; TAA, tumor-associated antigen; AYA, adolescents and young adults; TBL1, transducing b-like protein1; XPO1, exportin-1.

## Data Availability

No new data were created or analyzed in this study. Data sharing is not applicable to this article.
